# Management of *Bartonella* Prosthetic Valve Endocarditis without Cardiac Surgery

**DOI:** 10.3201/eid2305.161238

**Published:** 2017-05

**Authors:** Padmasayee Papineni, Aisling Carroll, Johannes Radvan, Carolyn Hemsley, John Chambers, Nicholas Cortes, Timothy Harrison, John L. Klein

**Affiliations:** St. Thomas' Hospital, London, UK (P. Papineni, C. Hemsley, J. Chambers, J.L. Klein);; University Hospital Southampton, Southampton, UK (A. Carroll);; Royal Bournemouth Hospital, Bournemouth, UK (J. Radvan);; Basingstoke and North Hampshire Hospital, Basingstoke, UK (N. Cortes);; Public Health England, London (T. Harrison)

**Keywords:** *Bartonella*, endocarditis, cardiac surgery, case study, prosthetic valve, bacteria

## Abstract

Two cases of *Bartonella* prosthetic valve endocarditis were cured when treated for 2 weeks with gentamicin and 3 months with doxycycline. Clinical cure correlated with decreased *Bartonella* antibody titers. This report suggests a strategy to monitor, treat, and cure *Bartonella* prosthetic valve endocarditis.

*Bartonella*, a causative agent of blood culture–negative endocarditis (BCNE) that usually requires valve surgery, was first attributed to endocarditis over 20 years ago (*1**–**4*). We report 2 cases of *Bartonella* prosthetic valve endocarditis that were successfully treated with antimicrobial drugs alone.

Case 1 involved a 62-year-old man admitted to the hospital in 2011 with left-flank pain and a 3-year history of anorexia, weight loss, night sweats, and recent diagnosis of anemia. He kept pet cats. He underwent mechanical aortic valve replacement and patent foramen ovale closure in 1992 and a repeat aortic valve replacement and an aortic root replacement for a chronic type A dissection in 1996. 

On physical examination, he was afebrile and had subconjunctival hemorrhages, normal prosthetic heart sounds, and 10-cm splenomegaly. Blood tests showed a hemoglobin concentration of 9 g/dL (reference range 14.0–17.5 g/dL), serum creatinine of 2.76 mg/dL (reference range 0.6–1.2 mg/dL), C-reactive protein of 48 mg/L (reference range 0.08–3.1 mg/L), rheumatoid factor of 742 U/mL (reference range 0–30 U/mL), and c-ANCA (cytoplasmic antineutrophil cytoplasmic antibody) positivity (proteinase 3 autoantibody concentration, 18.3 U/mL). Urine dipstick (Combur 7 Test; Roche Diagnostics Ltd, Basel, Switzerland) revealed the presence of blood (4+), and renal biopsy demonstrated necrotizing crescentic glomerulonephritis. Five blood cultures were sterile. Transesophageal echocardiography showed no evidence of endocarditis. *Bartonella* serologic testing was conducted with an indirect immunofluorescence assay by using the manufacturer’s instructions (MRL, Cypress, CA, USA); results showed high IgG titers to both *B. henselae* and *B. quintana* (Table), but the infecting species could not be determined. The patient was treated for 2 weeks with intravenous gentamicin (2 mg/kg/d) and for 3 months with oral doxycycline (100 mg 2×/d). Nine months after completing treatment, he was well: splenomegaly had resolved, and hemoglobin (15.6 g/dL) and creatinine (1.31 mg/dL) concentrations approached normal levels. He was well when last reviewed in 2014.

**Table:** *Bartonella* antibody titers in cases 1 and 2 by month after diagnosis*

Month after diagnosis	*B. henselae* IgM	*B. henselae* IgG	*B. quintana* IgM	*B. quintana* IgG
Case 1				
0	40	32,768	<20	1,024
8	<20	16,385	<20	512
11	20	8,096	<20	512
16	<20	8,096	<20	256
25	<20	4,096	<20	64
43	<20	2,048	<20	64
Case 2				
0	<20	8,192	<20	512
16	<20	≥512	<20	64
41	<20	64	<20	<64

*All time points were assayed in parallel. Antibody titers are the inverse of the greatest dilution that exhibited a reaction.

Case 2 involved a 29-year-old woman with inflammatory bowel disease and primary sclerosing cholangitis. Her symptoms began in 2011 with fever, rigors, night sweats, and anorexia for 2 weeks. She had no pets but recalled contact with a kitten 8 months previously. In 2002, BCNE developed, requiring mechanical aortic and mitral valve replacements. In 2003, BCNE was again diagnosed but was complicated by an ascending aorta to left atrial fistula, requiring an aortic root replacement, a homograft, and a repeat mechanical mitral valve replacement. 

Physical examination revealed fever, a splinter hemorrhage, and an ejection systolic murmur. Blood tests revealed a low hemoglobin concentration (10.9 g/dL), a high C-reactive protein concentration (26 mg/L), normal renal function, and positivity for rheumatoid factor (114 U/mL). Transesophageal echocardiograms revealed no evidence of endocarditis. One of 20 blood culture tests grew *B. henselae* after a 19-day incubation (Public Health England, identified by partial sequencing of 16S rDNA). A serologic test for *Bartonella* was strongly positive (Table). Three days after starting treatment with oral doxycycline (100 mg 2×/d) and intravenous gentamicin (3 mg/kg/d), she became afebrile; she received 14 days of gentamicin and 3 months of doxycycline in total. Two months later, she remained well, and her C-reactive protein concentration was <5 mg/L. Because of symptomatic stenosis caused by structural deterioration of the replacement aortic valve, she underwent another aortic valve and root replacement 19 months after completing antimicrobial drugs. No evidence of active endocarditis was found during surgery. 

*Bartonella* antibody titers dropped slowly over a period of 3 years in both patients (Table). Only case 1 had definite infective endocarditis when using the modified Duke diagnostic criteria. However, because the Duke criteria are insensitive for BCNE diagnosis, it has been proposed that a *Bartonella* IgG titer of >1:800 and a positive Western blot or PCR analysis when using valve or blood specimens should be considered major Duke criteria (*5*)*.*

Most reported cases of *Bartonella* endocarditis involve native valves; the first prosthetic valve infection was reported in 2002 (*6*). Although >80% of patients require valve replacement, infection with *Bartonella* is not in itself a recognized indication for surgery. Because our patients responded to medication, we did not need to consider cardiac surgery. The optimal antimicrobial drug therapy and duration for *Bartonella* endocarditis is undetermined. The recommended regimen of gentamicin for 14 days and doxycycline for 4 weeks (*7*) has limited evidence supporting its use (*8**,**9*). We found only 1 case of *Bartonella* prosthetic valve endocarditis cured without valve surgery; it was cured with a 30-month antimicrobial drug regimen (*10*)*.*

The role for serial serologic testing in assessing cure of *Bartonella* endocarditis is unknown. In our cases, as in a previous report (*10*), a drop in *Bartonella* titers occurred over a 3-year period in those who were cured, suggesting follow-up serologic testing might be useful to assess *Bartonella* endocarditis clinical cure.

Our findings suggest that a simple, inexpensive drug regimen is optimal therapy for *Bartonella* endocarditis and that serial serologic testing can confirm adequate treatment and cure. Further research is needed to validate this approach to managing *Bartonella* endocarditis.
